# ^18^F–Sodium Fluoride Uptake in Abdominal Aortic Aneurysms: The SoFIA^3^ Study

**DOI:** 10.1016/j.jacc.2017.11.053

**Published:** 2018-02-06

**Authors:** Rachael O. Forsythe, Marc R. Dweck, Olivia M.B. McBride, Alex T. Vesey, Scott I. Semple, Anoop S.V. Shah, Philip D. Adamson, William A. Wallace, Jakub Kaczynski, Weiyang Ho, Edwin J.R. van Beek, Calum D. Gray, Alison Fletcher, Christophe Lucatelli, Aleksander Marin, Paul Burns, Andrew Tambyraja, Roderick T.A. Chalmers, Graeme Weir, Neil Mitchard, Adriana Tavares, Jennifer M.J. Robson, David E. Newby

**Affiliations:** a British Heart Foundation Centre for Cardiovascular Science, University of Edinburgh, Edinburgh, United Kingdom; b Edinburgh Imaging Facility, Queen’s Medical Research Institute, University of Edinburgh, Edinburgh, United Kingdom; c National Health Service Lothian, Royal Infirmary of Edinburgh, Edinburgh, United Kingdom

**Keywords:** abdominal aortic aneurysm, positron emission tomography, repair, rupture, AAA, abdominal aortic aneurysm, CI, confidence interval, CT, computed tomography, FDG, fluorodeoxyglucose, MDS, most diseased segment, PET, positron emission tomography, SUV, standardized uptake value, TBR, tissue-to-background ratio, USPIO, ultrasmall superparamagnetic particles of iron oxide

## Abstract

**Background:**

Fluorine-18–sodium fluoride (^18^F-NaF) uptake is a marker of active vascular calcification associated with high-risk atherosclerotic plaque.

**Objectives:**

In patients with abdominal aortic aneurysm (AAA), the authors assessed whether ^18^F-NaF positron emission tomography (PET) and computed tomography (CT) predicts AAA growth and clinical outcomes.

**Methods:**

In prospective case-control (n = 20 per group) and longitudinal cohort (n = 72) studies, patients with AAA (aortic diameter >40 mm) and control subjects (aortic diameter <30 mm) underwent abdominal ultrasound, ^18^F-NaF PET-CT, CT angiography, and calcium scoring. Clinical endpoints were aneurysm expansion and the composite of AAA repair or rupture.

**Results:**

Fluorine-18-NaF uptake was increased in AAA compared with nonaneurysmal regions within the same aorta (p = 0.004) and aortas of control subjects (p = 0.023). Histology and micro-PET-CT demonstrated that ^18^F-NaF uptake localized to areas of aneurysm disease and active calcification. In 72 patients within the longitudinal cohort study (mean age 73 ± 7 years, 85% men, baseline aneurysm diameter 48.8 ± 7.7 mm), there were 19 aneurysm repairs (26.4%) and 3 ruptures (4.2%) after 510 ± 196 days. Aneurysms in the highest tertile of ^18^F-NaF uptake expanded 2.5× more rapidly than those in the lowest tertile (3.10 [interquartile range (IQR): 2.34 to 5.92 mm/year] vs. 1.24 [IQR: 0.52 to 2.92 mm/year]; p = 0.008) and were nearly 3× as likely to experience AAA repair or rupture (15.3% vs. 5.6%; log-rank p = 0.043).

**Conclusions:**

Fluorine-18-NaF PET-CT is a novel and promising approach to the identification of disease activity in patients with AAA and is an additive predictor of aneurysm growth and future clinical events. (Sodium Fluoride Imaging of Abdominal Aortic Aneurysms [SoFIA^3^]; NCT02229006; Magnetic Resonance Imaging [MRI] for Abdominal Aortic Aneurysms to Predict Rupture or Surgery: The MA3RS Trial; ISRCTN76413758)

Abdominal aortic aneurysm (AAA) disease affects up to 5% of men aged 64 to 75 years, and its prevalence is increasing in more elderly populations (1). With progressive AAA expansion over time, there is an increasing risk for often fatal rupture, representing the 12th commonest cause of death among older men (2). Consequently, patients with AAA enter an ultrasound-based surveillance program, with the aim of facilitating pre-emptive elective aneurysm repair to avoid fatal rupture. AAA surveillance relies on serial measurements of aneurysm diameter, which is currently the best clinical predictor of further expansion and rupture 3, 4. However, AAA growth is nonlinear, unpredictable, and influenced by biomechanical processes that cannot be predicted by conventional anatomic imaging alone (5). Indeed, aneurysms not infrequently rupture below the current threshold (55 mm in diameter) for elective repair, and many patients with aneurysms >70 mm never experience rupture (6). There is therefore a need to develop more reliable methods to identify patients who are at particular risk for AAA expansion and rupture (7).

In AAA disease, degradation of the extracellular matrix occurs in response to the accumulation of inflammatory cells, such as macrophages and lymphocytes, and the activation of matrix metalloproteinases. The resulting milieu of cellular inflammation, tissue destruction, and necrosis can lead to cycles of further inflammation (8). Focal “hotspots” of such intense biological activity have been identified in active aneurysm disease and can occur at the site of rupture (9). We have recently demonstrated that the positron-emitting radiotracer ^18^F–sodium fluoride (^18^F-NaF) can identify areas of early microcalcification (10) that occur in response to necrotic inflammation in ruptured or high-risk human carotid (11) and coronary (12) atherosclerotic plaques. This tracer has not been assessed in patients with AAA, although loss of tissue integrity and necrotic inflammation may be central to its pathophysiology, underlie aneurysm expansion, and ultimately predict disease progression and outcome (7). We hypothesized that ^18^F-NaF uptake on positron emission tomography (PET) would highlight areas of microcalcification and AAA disease activity, representing regions prone to expansion and rupture. The main aims of this study were to determine whether ^18^F-NaF uptake on combined PET and computed tomography (CT) is increased in AAA and whether this is associated with aneurysm growth (the primary endpoint) and subsequent rates of AAA repair or rupture.

## Methods

### Study population

Consecutive patients older than 50 years under routine clinical surveillance with asymptomatic AAA (≥40 mm anteroposterior diameter) were recruited from the MA3RS (Magnetic Resonance Imaging in Abdominal Aortic Aneurysms to Predict Rupture or Surgery) study (ISRCTN76413758) database (13). Control subjects were recruited through the National Health Service Lothian National Abdominal Aortic Aneurysm Screening Programme or the Vascular Laboratory at the Royal Infirmary of Edinburgh and had documented normal-caliber aortas (<30-mm anteroposterior diameter).

### Study design

This was a prospective single-center, case-control, observational cohort study of patients with asymptomatic AAA who were under ultrasound-based surveillance as part of routine clinical follow-up and control subjects with normal-caliber abdominal aortas demonstrated on targeted screening ultrasound. The study (NCT02229006) was conducted with the written informed consent of all subjects, with approval by the research ethics committee, and in accordance with the Declaration of Helsinki.

### Study assessments

Participants underwent a clinical evaluation including documentation of medical history, concomitant medications, and family history as well as an ultrasound evaluation of the maximum anteroposterior abdominal aortic diameter. Ultrasound scans were carried out in an accredited clinical vascular science laboratory using a standardized protocol with known interobserver variability of 3.4% (14). The AAA growth rate was determined using the AAA maximum anteroposterior diameter obtained at baseline and the last ultrasound examination performed during study follow-up. Abdominal aortic tissue was obtained at postmortem or from patients undergoing elective AAA repair and analyzed by micro–PET-CT and histology (Online Appendix).

### ^18^F-NaF PET-CT

**Figure 1:**
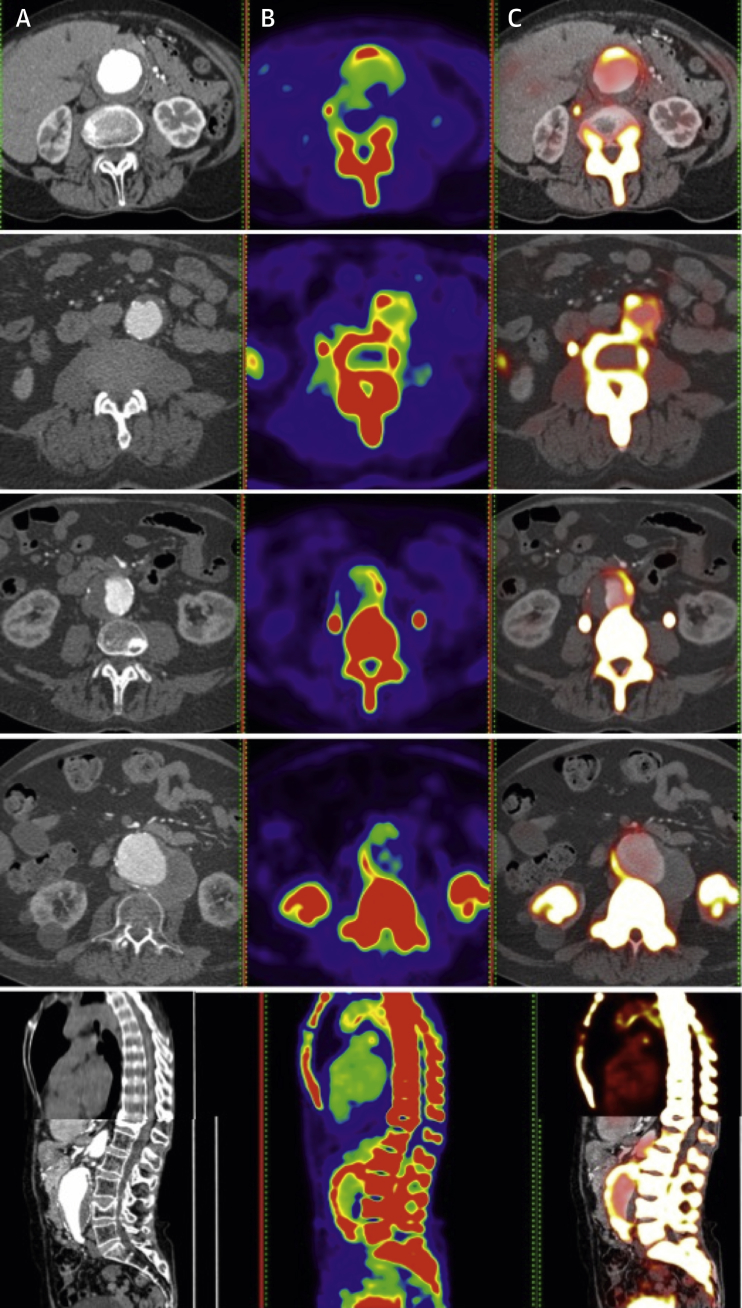
Positron Emission Tomographic and Computed Tomographic Images of Abdominal Aortic Aneurysms **(A)** Structural image of computed tomographic angiography, **(B)**^18^F–sodium fluoride uptake on positron emission tomography, and **(C)** fused positron emission tomographic–computed tomographic images colocalizing ^18^F–sodium fluoride uptake with the skeleton and abdominal aortic aneurysm.

Patients were administered a target dose of 125 MBq of ^18^F-NaF intravenously and after 60 min were imaged on a hybrid 128–detector array PET-CT scanner (Biograph mCT, Siemens Healthcare, Erlangen, Germany) (10). A low-dose attenuation correction CT scan was performed (120 kV, 50 mAs, 5/3 mm), followed by acquisition of PET data, using 3 10-min bed positions to ensure coverage from the thoracic aorta to the aortic bifurcation. An electrocardiographically gated calcium scoring CT scan (120 kV, 120 mAs, 3/3 mm; prospective electrocardiographic gating at 50% of the R-R interval) and contrast-enhanced CT angiography (120 kV, 145 mAs, 3/3 mm, field of view 400; and 1/1 mm, field of view 300; triggered at 181 Hounsfield units) were performed, centered on the AAA (or abdominal aorta in control patients) and extending to the aortic bifurcation (Figure 1).

To estimate ^18^F-NaF uptake, the maximum standardized uptake values (SUVs) (a validated measure of tissue radiotracer uptake) were quantified from regions of interest (Online Appendix) (15). Maximum tissue-to-background ratios (TBRs) were then calculated, after correction for blood pool activity using the averaged mean SUVs of 3 consecutive regions of interest from the right atrium, according to our previously described technique (16). Although TBR_max_ was used for our primary analysis (12), we also investigated other methods for quantification, including SUV_max_ and corrected SUV_max_ (calculated by subtracting the blood pool activity from SUV_max_) (17). Finally, we adopted the “most diseased segment” (MDS) approach, as suggested by others 11, 16, 18, 19, 20. The MDS TBR_max_ was calculated as the average TBR_max_ across 3 axial slices centered on the region of the aneurysm with the highest tracer activity (11).

### Clinical endpoints and adjudication

Clinical data from clinic visits, the research database, electronic health records, primary care contacts, and the General Register Office were reviewed and clinical endpoints adjudicated by the independent Clinical Endpoint Committee (Online Appendix). The committee members were blinded to the findings of PET-CT. Follow-up was censored at January 10, 2017, or at the time of event.

### Statistical and data analysis

Baseline characteristics are reported as number (percentage) for categorical variables and as mean ± SD or median (interquartile range) for continuous variables, as appropriate. We stratified our patient cohort by AAA MDS TBR_max_ tertiles to assess associations with aneurysm expansion (the primary endpoint) and the clinical outcomes of aneurysm repair or rupture (the secondary endpoint). For the aneurysm growth and outcomes analysis, AAA expansion rate and MDS TBR_max_ were log-transformed (log_2_) to normalize the data. One-way analysis of variance was used to compare continuous data across multiple factors, with post hoc analysis using the Bonferroni test as appropriate. The Kruskal-Wallis test was used for nonparametric continuous data and the log-rank test for comparisons of AAA event rate and mortality between tertiles. Categorical data were compared using chi-square or Fisher exact tests, and the unpaired Student’s *t*-test was used to compare continuous outcomes between 2 independent groups. Two-tailed Pearson correlation and linear regression analysis were performed to investigate the relationship between ^18^F-NaF uptake and aneurysm expansion. Finally, we performed Kaplan-Meier and Cox regression analysis to investigate time to AAA event by tertile, censored at the date of death. Statistical analysis was undertaken using SPSS Statistics 23 (IBM, Armonk, New York), and significance was taken at the 2-sided 5% level (p < 0.05).

## Results

**Figure 2:**
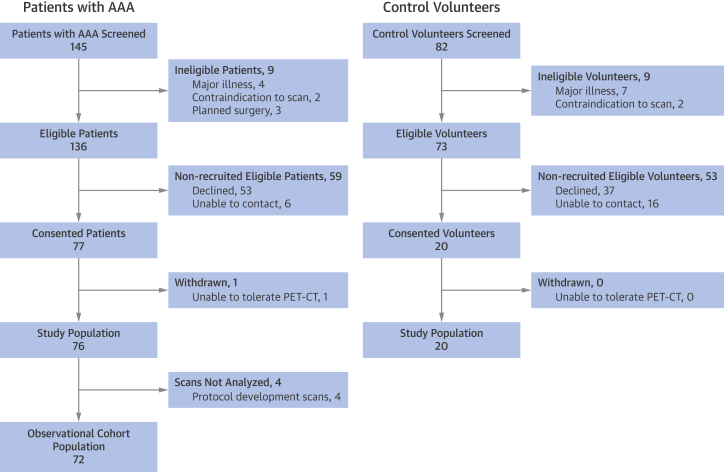
Study Populations The 20 patients for the case-control study were selected from within the cohort study population. AAA = abdominal aortic aneurysm; CT = computed tomography; PET = positron emission tomography.

A total of 145 patients with AAA were screened for inclusion: 136 were approached, and 76 patients ultimately attended for the scanning visit. Four patients underwent protocol development scans, had incomplete data, and were excluded from the final analysis, leaving a total of 72 patients with AAA and 20 control subjects (Figure 2). Patients were predominantly elderly (mean age 72.5 ± 6.9 years) men (84.7%) with multiple cardiovascular risk factors, including hypertension (65.3%) and hypercholesterolemia (81.9%) (Table 1). More than 90% were current or ex-smokers (27.8% and 65.3%, respectively), with a mean baseline AAA diameter of 48.8 ± 7.7 mm. Control subjects were younger (mean age 65.2 ± 2.8 years) but also predominantly men (95.0%), and 40% were current (25%) or prior (15%) smokers.

**Table 1:** Characteristics of Study Participants

	Cohort Study					Case-Control Study	
	All Patients With AAA (N = 72)	Tertile 1 (n = 24)	Tertile 2 (n = 24)	Tertile 3 (n = 24)	p Value∗	Patients With AAA (n = 20)	Control Subjects (n = 20)
Characteristics							
Age, yrs	72.5 ± 6.9	73.3 ± 7.2	72.8 ± 7.5	71.4 ± 6.1	0.640	66.2 ± 2.6	65.2 ± 2.8
Male	61 (84.7)	20 (83.3)	21 (85.7)	20 (83.3)	1.000	19 (95.0)	19 (95.0)
Systolic blood pressure, mm Hg	136.7 ± 18.3	142.1 ± 18.2	132.5 ± 16.2	135.6 ± 19.9	0.178	138.1 ± 22.5	141.6 ± 14.2
Diastolic blood pressure, mm Hg	81.6 ± 11.6	84.8 ± 12.0	76.4 ± 9.4	83.5 ± 11.9	0.024	84.2 ± 16.6	80.5 ± 8.2
Heart rate, beats/min	71 ± 9	72 ± 10	70 ± 8	70 ± 8	0.694	70.2 ± 9.7	66.7 ± 13.5
Body mass index, kg/m^2^	27.6 ± 3.5	27.6 ± 3.4	26.2 ± 3.3	29.0 ± 3.3	0.019	28.4 ± 3.1	29.3 ± 6.4
Current smoker	20 (27.8)	6 (25.0)	9 (37.5)	5 (20.8)	0.407	5 (25.0)	5 (25.0)
Medical history							
Hypertension	47 (65.3)	14 (58.3)	16 (66.7)	17 (70.8)	0.651	12 (60.0)	6 (30.0)
Hypercholesterolemia	59 (81.9)	21 (87.5)	21 (87.5)	17 (70.8)	0.264	15 (75.0)	7 (35.0)
Diabetes	10 (13.9)	3 (12.5)	4 (16.7)	3 (12.5)	1.000	1 (5.0)	2 (10.0)
Ischemic heart disease	22 (30.6)	7 (29.2)	7 (29.2)	8 (33.3)	0.937	5 (25.0)	1 (5.0)
Peripheral arterial disease	11 (15.3)	2 (8.3)	8 (33.3)	1 (4.2)	0.021	2 (10.0)	1 (5.0)
Cerebrovascular disease	10 (13.9)	1 (4.2)	4 (16.7)	5 (20.8)	0.316	1 (5.0)	0 (0.0)
Positive family history of AAA	9 (12.5)	2 (8.3)	4 (16.7)	3 (12.5)	0.903	3 (15.0)	2 (10.0)
Medications							
Antiplatelet agents	51 (70.8)	19 (79.2)	18 (75.0)	14 (58.3)	0.350	11 (55.0)	3 (15.0)
Statins	58 (80.6)	21 (87.5)	21 (87.5)	16 (66.7)	0.141	13 (65.0)	8 (40.0)
Anticoagulant agents	2 (2.8)	1 (4.2)	0 (0.0)	1 (4.2)	1.000	0 (0.0)	1 (5.0)
Beta-blockers	19 (26.4)	8 (33.3)	5 (20.8)	6 (25.0)	0.711	6 (30.0)	2 (10.0)
ACE inhibitors	25 (34.7)	8 (33.3)	8 (33.3)	9 (37.5)	1.000	5 (25.0)	2 (10.0)
Aorta							
Aortic diameter, mm	48.8 ± 7.7	47.5 ± 9.2	48.7 ± 7.8	50.1 ± 5.8	0.510	45.7 ± 4.0	17.6 ± 2.3
Concurrent iliac aneurysm	13 (18.1)	4 (16.7)	5 (20.8)	4 (16.7)	1.000	3 (15.0)	0.0 (0.0)

Values are mean ± SD or n (%).

AAA = abdominal aortic aneurysm; ACE = angiotensin-converting enzyme; ^18^F-NaF = ^18^F–sodium fluoride.

∗ p value for trend across the tertiles.

### Case-control study

Twenty patients with AAA were matched for age, sex, and smoking status with the 20 control subjects (Table 1). Background blood pool activity in the right atrium was similar between groups (log_2_ SUV_mean_ −0.570 ± 0.517 vs. −0.588 ± 0.531; difference 0.018; 95% confidence interval [CI]: −0.340 to 0.376; p = 0.919). Fluorine-18-NaF uptake was higher in the AAA when compared with the abdominal aorta of control subjects irrespective of the method of quantification (e.g., log_2_ MDS TBR_max_ 1.712 ± 0.560 vs. 1.314 ± 0.489; difference 0.398; 95% CI: 0.057 to 0.739; p = 0.023) (Online Table 1). In contrast to control aortic tissue, AAA tissue demonstrated ex vivo ^18^F-NaF uptake that correlated with areas of tissue disruption with necrotic debris and active calcification (r = 0.808, p = 0.015) (Figure 3). Areas of ^18^F-NaF uptake on PET were distinct from areas of macrocalcification on CT (Online Video 1).Figure 3Correlation of Histology With Micro–Positron Emission Tomography and Computed Tomography of Abdominal Aortic TissueEx vivo micro–positron emission tomography and computed tomography **(left)** and histology **(right)** of aortic wall excised **(A)** at postmortem in a patient without an aneurysm and **(B)** during open abdominal aortic aneurysm repair. Regions of interest **(dashed circle)** of ^18^F–sodium fluoride (^18^F-NaF) uptake demonstrate atheromatous disease with necrosis (hematoxylin and eosin stain, magnification ×100 [Online Video 1]; **B1**) and calcification (**black**, Von Kossa stain, magnification ×200; **B2**) in the aortic aneurysm tissue that is not apparent in control aorta **(A1, A2)**.
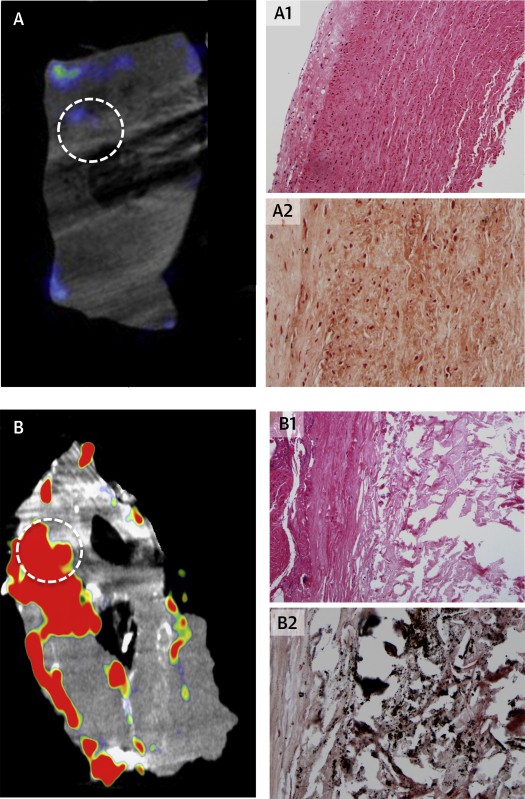

**Figure:**
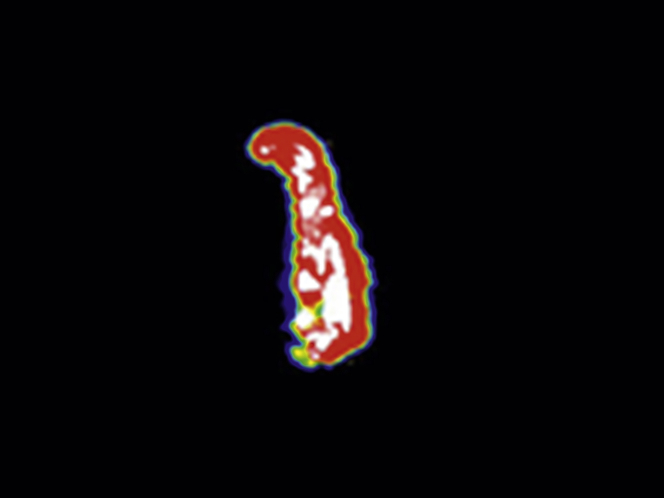
Online Video 1 Micro-positron emission tomography and computed tomography of aortic aneurysm tissue following *ex vivo* incubation with ^18^F-sodium fluoride. Note ^18^F-sodium fluoride uptake (red) by positron emission tomography is distinct from the macrocalcification (white) identified with computed tomography. Some areas of macrocalcification show little or no associated ^18^F-sodium fluoride whereas other areas have avid ^18^F-sodium fluoride uptake in the absence of macrocalcification.

Patients with AAA had more cardiovascular risk factors and higher abdominal aortic CT calcium scores (log_2_ Agatston score 11.444 ± 1.760 vs. 7.338 ± 3.811; difference 4.105; 95% CI: 2.013 to 6.198; p = 0.001) (Online Table 1) than control subjects. However, no differences in ^18^F-NaF uptake were observed between these groups in either the descending thoracic aorta or the nonaneurysmal abdominal aorta.

### Observational cohort study

Fluorine-18-NaF uptake was again higher in the aneurysm than in the nonaneurysmal portion of the abdominal aorta (log_2_ TBR_max_ 1.647 ± 0.537 vs. 1.332 ± 0.497; difference 0.314; 95% CI: 0.0685 to 0.560; p = 0.004) and almost double the uptake observed in the descending thoracic aorta (log_2_ TBR_max_ 1.647 ± 0.537 vs. 0.881 ± 0.414; difference 0.766; 95% CI: 0.517 to 1.011; p < 0.0001). These differences were consistently observed regardless of the method of PET quantification.

Across the tertiles of AAA MDS TBR_max_, there were no differences in most risk factors for AAA disease, including age, sex, smoking habit, aneurysm diameter, hypertension, and hypercholesterolemia. Although there appeared to be some differences with respect to diastolic blood pressure, body mass index, and peripheral arterial disease, the trend was inconsistent across the tertiles (Table 1).

### ^18^F-NaF uptake and aneurysm growth

**Figure 4:**
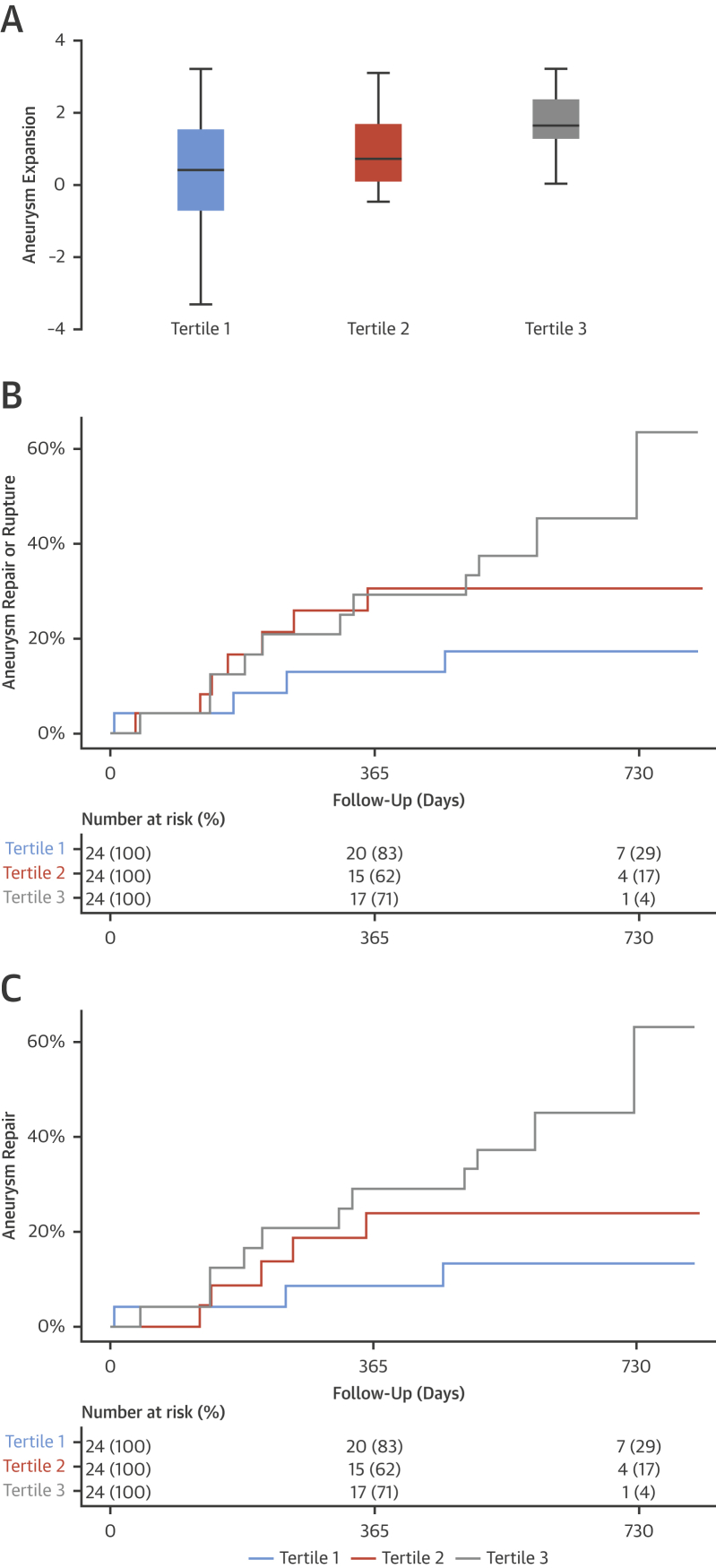
Prediction of Disease Progression and Clinical Outcome by ^18^F–Sodium Fluoride Positron Emission Tomography Association of ^18^F–sodium fluoride (^18^F-NaF) uptake with disease progression and clinical outcome. **(A)** Rate of aneurysm expansion (millimeters per year, log_2_ transformed) across the tertiles of ^18^F-NaF uptake. The highest tertile expanded more rapidly than those in the lowest tertile (3.10 vs. 1.24 mm/year, respectively, p = 0.008). Cumulative event rate (censored at date of death) across the tertiles of ^18^F-NaF uptake for **(B)** abdominal aortic aneurysm repair or rupture (log-rank p = 0.043) and **(C)** abdominal aortic aneurysm repair (log-rank p = 0.014).

During 510 ± 196 days of follow-up, the median AAA expansion rate was 2.20 mm/year (interquartile range: 0.96 to 3.72 mm/year) (Table 2). Baseline ^18^F-NaF activity in the aneurysm was associated with future expansion regardless of the method of quantification (e.g., log_2_ MDS TBR_max_ r = 0.365; p = 0.006). When stratified by tertiles, aneurysms in the highest tertile expanded 2.5× more rapidly than those in the lowest tertile (3.10 mm/year [IQR: 2.34 to 5.92 mm/year] vs. 1.24 mm/year [IQR: 0.52 to 2.92 mm/year]; p = 0.008) (Figure 4). Moreover, in multivariate analysis, ^18^F-NaF activity in the AAA (MDS TBR_max_) emerged as a predictor of growth independent of age, sex, baseline diameter, body mass index, blood pressure, smoking, renal function, or peripheral arterial disease (p = 0.042) (Online Table 2). In contrast, the aneurysm Agatston score was not associated with future expansion (r = 0.199; p = 0.141).

**Table 2:** Expansion Rate and Clinical Outcomes According to Tertiles of ^18^F–Sodium Fluoride Uptake

Outcome	All Patients With AAA (N = 72)	Tertile 1 (n = 24)	Tertile 2 (n = 24)	Tertile 3 (n = 24)	p Value∗
AAA expansion rate, mm/yr	2.20 (0.96–3.73)	1.24 (0.52–2.92)	1.55 (0.81–3.12)	3.10 (2.34–5.92)	0.008
AAA events					
Composite events	22 (30.6)	4 (16.7)	7 (29.2)	11 (45.8)	0.043
Repair	19 (26.4)	3 (12.5)	5 (20.8)	11 (45.8)	0.014
Rupture	3 (4.2)	1 (4.2)	2 (8.3)	0 (0.0)	
Deaths					
All-cause	8 (11.1)	4 (16.7)	4 (16.7)	0 (0.0)	0.343
AAA-related	3 (4.2)	1 (4.2)	2 (8.3)	0 (0.0)	—

Values are median (interquartile range) or n (%).

AAA = abdominal aortic aneurysm.

∗ p value for trend across the tertiles.

### ^18^F-NaF uptake and clinical events

In total, 22 patients (30.6%) met the composite endpoint of AAA repair or rupture. Of these, 19 (26.4%) underwent elective AAA repair and 3 (4.2%) experienced AAA rupture, all of whom died without repair. Five other patients died during study follow-up, all from non-AAA causes.

Patients with aneurysms in the highest tertile of ^18^F-NaF uptake were more likely to experience AAA repair or rupture during follow-up (15.3% vs. 5.6%; log-rank p = 0.043) (Table 2). They also had a reduced time to AAA event: 572 days versus 735 days for AAA repair (log-rank p = 0.014) and 572 days versus 709 days for the composite of AAA repair or rupture (log-rank p = 0.043) (Figure 4). In those patients who experienced AAA events, ^18^F-NaF activity was higher than in those who continued under surveillance without events (log_2_ MDS TBR_max_ 2.20 ± 0.58 vs. 1.87 ± 0.54; difference 0.330; 95% CI: 0.047 to 0.613; p = 0.023). In unadjusted analysis, a doubling of ^18^F-NaF activity in the MDS was associated with a more than 2-fold risk for experiencing AAA rupture or repair (hazard ratio: 2.16; 95% CI: 1.03 to 4.50; p = 0.041) (Table 2). When adjusted for age, sex, baseline diameter, systolic blood pressure, body mass index, and smoking, this risk remained (hazard ratio: 2.49; 95% CI: 1.07 to 5.78; p = 0.034) (Online Table 2).

## Discussion

In this prospective series of clinical studies, we have demonstrated for the first time that ^18^F-NaF uptake is specifically increased in AAA and relates to areas of advanced aneurysmal disease. Moreover, ^18^F-NaF uptake is a major predictor of aneurysm expansion and clinical outcome that is additive to standard clinical risk factors, including aneurysm diameter. This is the first study to demonstrate that an imaging biomarker of disease activity can add to the risk prediction of AAA and to suggest that this approach might refine clinical decisions regarding the need for surgery and improve patient outcomes (Central Illustration).

**Central Illustration:**
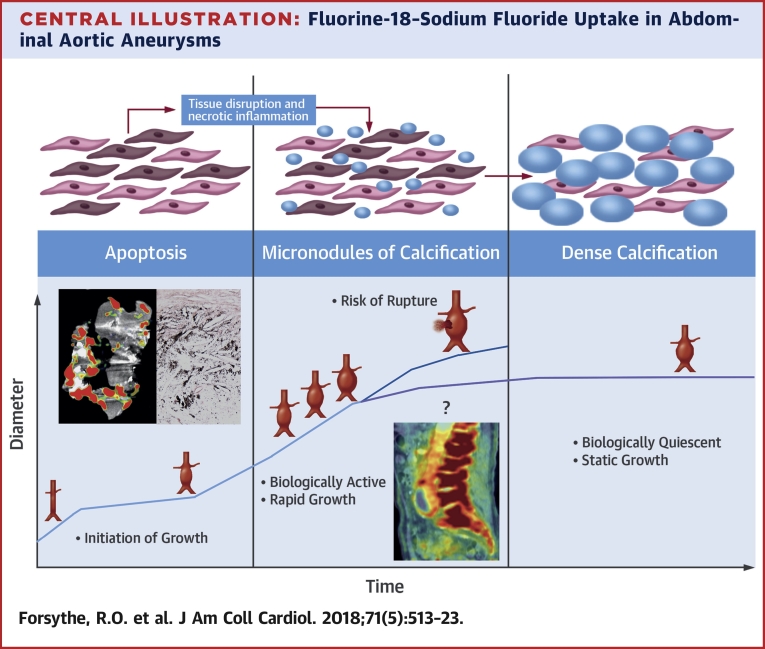
Fluorine-18–Sodium Fluoride Uptake in Abdominal Aortic Aneurysms Fluorine-18–sodium fluoride uptake is specific to abdominal aortic aneurysm tissue, is proportional to the rate of aneurysm expansion, and predicts the risk for repair or rupture independent of aneurysm diameter.

Our studies have several major strengths and prominent observations. First, we have shown AAA tissue demonstrates markedly increased levels of ^18^F-NaF uptake that far exceed those seen in control volunteers. Perhaps more important, uptake of the AAA also exceeds that observed in the nonaneurysmal aorta within the same patient. Second, we demonstrate that ^18^F-NaF uptake localized to areas of AAA disease, highlighting diseased areas of poor tissue integrity that may be susceptible to aneurysm expansion and clinical events. Third, we assessed the potential clinical value of this technique in a cohort of patients with extended follow-up in which the clinicians responsible for the patient’s care were unaware of the findings of PET-CT. It is therefore salient to note that ^18^F-NaF uptake predicted expansion and clinical outcomes in addition to clinical risk factors including AAA diameter, especially as the latter drives the decision for elective AAA repair. Fourth, this is the largest dedicated study using PET-CT in AAA disease to date and the first clinical study to investigate ^18^F-NaF PET-CT in AAA disease progression (21). Finally, this was a prospective clinical cohort study, in contrast with many previous studies of PET-CT in patients with AAA that are based on retrospective data, often obtained from cohorts derived from oncological imaging practice.

We previously demonstrated that ^18^F-NaF selectively binds to microcalcification in coronary (11) and carotid atherosclerotic 10, 11 plaques and that this is associated with plaque vulnerability and rupture. We 11, 12 and others (22) have also shown that ^18^F-NaF binds to areas of tissue necrosis–associated myocardial and cerebral infarction. In our present study, data from histology and micro–PET-CT indicate that this tracer behaves in a similar fashion in AAA. Increased ^18^F-NaF uptake was most marked in AAA tissue with advanced disease and active calcification. We suggest that ^18^F-NaF uptake again relates to microcalcification and is particular to the most diseased areas associated with tissue disruption and loss of integrity. Interestingly, we also showed that ^18^F-NaF was distinct from AAA macrocalcification detected by CT and that the latter is not associated with expansion or AAA events, suggesting that once established, dense calcified deposits represent a more stabilized biological state.

Most previous clinical studies using PET-CT in AAA disease have focused on the use of ^18^F-fluorodeoxyglucose (FDG) to identify inflammation, with variable results and no clear clinical application. Although some groups have suggested a potential role for ^18^F-FDG PET-CT in predicting AAA expansion or rupture 23, 24, 25, others have disputed this and reported contradictory findings 26, 27. This in part relates to the small study sample sizes and whether patients had symptomatic or inflammatory AAAs, but to date there is no clear relationship between ^18^F-FDG and aneurysm expansion or clinical outcome (22). Our present study was nested within another larger clinical cohort study, the MA3RS trial. This was a multicenter study of 342 patients with AAA who underwent ultrasmall superparamagnetic particles of iron oxide (USPIO)–enhanced magnetic resonance imaging to identify cellular inflammation within the aortic wall (13). This study recently reported and demonstrated that although USPIO-enhanced magnetic resonance imaging did predict AAA expansion and clinical outcome, the association was modest and was not independent of established clinical factors, including ultrasound AAA diameter (28). This suggests that imaging of cellular inflammation alone, either by ^18^F-FDG or USPIO-enhanced magnetic resonance imaging, is insufficient to provide additive clinical information beyond established clinical risk factors and AAA diameter. In contrast, ^18^F-NaF PET-CT identifies focal areas of microcalcification indicative of more advanced aneurysm disease and independently predicts both disease progression and clinical events.

We have demonstrated an important clinical application of ^18^F-NaF PET-CT in AAA disease. Until now, future prediction of aneurysm expansion has relied on the simple morphological parameter of aneurysm diameter. However, it is clear that AAA growth is nonlinear and cannot be predicted accurately from a simple anatomic measure, such as AAA diameter. Although we know that larger aneurysms tend to expand more rapidly and are more prone to rupture, disease evolution is not straightforward. Better AAA disease prediction using ^18^F-NaF uptake could be particularly useful for patients in whom the decision to intervene is challenging, such as those with high-risk aneurysms smaller than 55 mm, those with borderline aneurysm sizes, and those with larger aneurysms where the balance of risk and benefit is uncertain.

### Study limitations

This was a single-center proof-of-concept study with a small number of rupture events, making adjustment for potential confounders and covariates challenging. Although we observed no marked differences in sex across the tertiles of ^18^F-NaF uptake, our study population had a strong male bias (typical of this disease population), and we cannot be certain that our findings are truly representative of both men and women. The clinical impact of this technique has not been assessed and would require a larger trial in which clinical and surgical decisions would be influenced or dictated by the findings of ^18^F-NaF PET-CT. The widespread implementation of this technique may be challenging, especially given the relative expense and complexity of PET-CT compared with ultrasound. However, we have demonstrated the feasibility of this technique, which uses a well-established, widely available, and relatively cheap radiotracer. Moreover, with the more widespread use and availability of PET-CT scanners, barriers to implementation are declining. There are also some inherent limitations of ^18^F-NaF image analysis that merit comment. Being a bone tracer, ^18^F-NaF is readily taken up by the vertebrae, which lie in close proximity to the abdominal aorta. In our study, it was necessary to exclude some areas of the posterior aorta because of overspill of signal. However, this is not unique to ^18^F-NaF, with similar issues seen with ^18^F-FDG uptake in regions of interest adjacent to bowel, muscle, or other metabolically active tissues. Finally, further validation of the tissue binding characteristics and time course of change in ^18^F-NaF uptake in aneurysmal and nonaneurysmal aortas are needed, and this would be interesting to explore in future studies.

## Conclusions

This novel proof-of-concept PET-CT study of patients with asymptomatic AAA demonstrates that ^18^F-NaF uptake identifies advanced aneurysmal disease and is associated with aneurysm growth and clinical AAA events independent of established clinical risk factors, including aneurysm diameter. This technique holds major promise for the future management of patients with AAA disease.

Perspectives **COMPETENCY IN MEDICAL KNOWLEDGE:**^18^F-Sodium fluoride uptake is specific to abdominal aortic aneurysm tissue, proportional to the rate of aneurysm expansion, and predictive of repair or rupture, independent of aneurysm diameter.

**TRANSLATIONAL OUTLOOK:** Additional studies are needed to clarify the clinical utility of this imaging biomarker as a guide to selection of patients for elective repair of abdominal aortic aneurysms.
