# Supplementary material for: 18F–Sodium Fluoride Uptake in Abdominal Aortic Aneurysms: The SoFIA3 Study
Source: J Am Coll Cardiol. 2018 Feb 6;71(5):513–23. doi: 10.1016/j.jacc.2017.11.053 (PMC5800891; doi:10.1016/j.jacc.2017.11.053)
Supplement: Online Data [file mmc1.docx]

**^SUPPLEMENTAL MATERIAL^**

**Supplemental Methods**

**Patient Population: Exclusion Criteria**

Participants were excluded if they were unable to give informed written consent, or had contraindications to iodine-based contrast media, estimated glomerular filtration rate <30 mL/min/1·73 m^2^, a collagen vascular disorder, an intercurrent illness, a life expectancy <1 year, or were women of child-bearing potential without contraception.

**Tissue Collection and Micro-Positron Emission Tomography**

In two patients, aneurysmal aortic tissue was obtained during open abdominal aortic aneurysm (AAA) repair and was excised from the anterior wall of the native aorta prior to plication around the tube graft. Non-aneurysmal (control) aortic tissue was obtained during post-mortem examination of a sudden cardiac death victim, following written consent from relatives and the approval of the Research Ethics Committee.

Aneurysmal and non-aneurysmal aortic tissues were incubated using optimized protocol conditions, specifically: 20-min incubation with ^18^F-sodium fluoride (100 kBq/mL, in 0.1 M phosphate buffer saline) at room temperature. Following incubation, samples were washed twice in assay buffer (1 min per wash) and mounted on a petri dish for micro-positron emission tomography (PET) and computed tomography (CT) imaging. This incubation protocol was designed to capture pseudo-secular equilibrium of ^18^F-sodium fluoride binding to calcium particles and aggregates in tissue, mimicking *in vivo* conditions, while minimizing tissue degradation during incubation process with the aim to retain tissue properties for subsequent histology. Incubation conditions were optimized using calcium phosphate particles (CPPs, 200-300 nm) and varying concentrations of ^18^F-sodium fluoride, as well as, incubation time with and without competing non-homologous inhibitor (alendronate 25 µM). In brief, results from those optimization experiments demonstrated that ^18^F-sodium fluoride total and specific binding to CPPs was rapid and reached pseudo-secular equilibrium at 20 min following incubation. The affinity, K_D_, of ^18^F-sodium fluoride to CPPs was 1 nM. For molar activity of ^18^F-sodium fluoride of 100 GBq/µmol, incubations total volume of 200 mL and activity of 20 MBq equals 100 kBq/mL of ^18^F-sodium fluoride (i.e. 1 nM).

Following incubation of the aortic specimen with ^18^F-sodium fluoride, a 30-min emission scan was obtained using a micro-PET-CT scanner (nanoPET/CT; Mediso, Hungary) in a 1:5 co-incidence mode. A CT scan was acquired (semi-circular full trajectory, maximum field of view, 720 projections, 55 kVp, 300 ms and 1:4 binning) for attenuation correction. PET data were reconstructed using Mediso’s iterative Tera-Tomo 3D reconstruction algorithm using 4 iterations, 6 subsets, full detector model, normal regularisation, spike filter on, voxel size 0.4 mm and 400-600 keV energy window.

**Histopathological analysis**

Regions of interest (ROI) were identified on the reconstructed micro-PET-CT images of the AAA and control aortic specimens. Specimens of tissue corresponding to the site of each ROI were stained with hemotoxylin and eosin to determine cellular structure, and Von Kossa to identify focal calcification.

**^18^F-NaF PET-CT Image Analysis**

Static PET-CT images were reconstructed with correction applied for attenuation, dead time, scatter and random coincidences, using an optimized iterative reconstruction algorithm (ultra-HD; TrueX + TOF, matrix 200, zoom 1; Gaussian filter). Scans were visually assessed for registration, patient movement, scan quality and tracer uptake, with minor adjustments in co-registration made manually as required, using fixed boney landmarks. PET analysis was performed using an OsiriX workstation (OsiriX, version 8·0·1 64 bit; OsiriX Imaging Software, Geneva, Switzerland). Care was taken to exclude tracer uptake that originated from nearby bone structures or from the urinary tract.

**CT Calcium Scoring**

CT Calcium scoring was performed by an experienced observer on a dedicated workstation (Vitrea Advanced, Vital Imaging, Toshiba Systems, Minnesota, USA). Taking care to exclude calcium originating from the vertebrae, the aortic calcium burden was extracted using axial slices of the ECG-gated calcium scoring CT and recorded as the Agatston score. The cumulative Agatston score was used to determine the macroscopic calcium burden for each region of the aorta: descending thoracic aorta, non-aneurysmal abdominal aorta and the aneurysm (or infra-renal abdominal aorta in control subjects) using a threshold of 130 Hounsfield Units.

**Clinical Endpoint Adjudication**

There are no consensus guidelines to direct investigators when adjudicating endpoints relating to death in patients with known abdominal aortic aneurysms who have no definitive proof of causality (such as post-mortem examination). Most of the recently proposed classification systems relating to abdominal aortic aneurysms events require radiological or operative evidence to classify abdominal aortic aneurysms events. In the event that no such confirmation was available, the end points were assessed and determined by each member of the end-point committee and any disagreement settled by consensus, with oversight from the Chairperson. ‘Cold pursuit’ of relevant data included obtaining information from primary care or hospital records as to likely cause of death, cause of death listed on death certificate obtained from public registry, and data from the Information and Statistics Division of NHS Scotland. The following guidelines and classification system were used as part of the assessment for adjudication of cause of death:

***Abdominal Aortic Aneurysms-related Events***

1. Abdominal aortic aneurysm-related death, confirmed
   - Death occurs in hospital following confirmed abdominal aortic aneurysm rupture on computed tomography, or intra-operative findings
   - Death occurs following treatment for abdominal aortic aneurysm during same admission (elective or emergency)
   - Death arising from complications relating to abdominal aortic aneurysm treatment
   - Death occurs out of hospital, confirmed abdominal aortic aneurysm rupture on post-mortem
   - Death occurs out of hospital, confirmed abdominal aortic aneurysm treatment related complication on post-mortem
2. Abdominal aortic aneurysm-related death, probable

- Death occurs in circumstances with high probability of abdominal aortic aneurysm rupture (e.g. patient admitted to hospital following collapse, known abdominal aortic aneurysm, hypotension, no other obvious cause of symptoms, death shortly after presentation)
- Death occurs in circumstances with high probability of complications relating to abdominal aortic aneurysm treatment (e.g. known endoleak, suspected rupture post-endovascular aortic repair)
- Death occurs out of hospital, no post-mortem performed but highly likely as no other obvious cause of death (e.g. witnessed collapse at home, known abdominal aortic aneurysm, sudden death, no other obvious cause)
- Sudden unexplained death in a patient with known abdominal aortic aneurysm ≥7 cm, with no other cause of death identified

1. Abdominal aortic aneurysm-related death, possible
   - Death occurs in hospital, no post-mortem performed and cannot be judged as highly likely to be abdominal aortic aneurysm-related, but no other circumstances to suggest other definitive cause
   - Death occurs out of hospital, no post-mortem performed and cannot be judged as highly likely to be abdominal aortic aneurysm-related, but no other circumstances to suggest other definitive cause

***Non-abdominal Aortic Aneurysm related events***

Cardiovascular death (non-abdominal aortic aneurysm):

Death resulting from an acute myocardial infarction, sudden cardiac death, death due to heart failure, death due to stroke, death due to cardiovascular procedures, death due to other cardiovascular causes (excluding abdominal aortic aneurysm-related deaths).

Non-cardiovascular death:

Death resulting from all other causes, excluding cardiovascular or abdominal aortic aneurysm-related deaths.

**Supplemental Table 1**

^18^F-Sodium Fluoride Uptake and Agatston Score in Patients with Abdominal Aortic Aneurysm and Control Subjects

| **Region** | **Uptake Measure [log_2_]** | **Population** | |  | |
| --- | --- | --- | --- | --- | --- |
|  |  | **Patients with AAA**  (n=20) | **Control Subjects**  (n=20) | **Mean difference**  **(95% CI for difference)** | **P-value** |
| Right atrium | | | | | |
|  | SUV_mean_ | -0·570±0·571 | -0·588±0·531 | 0·018 (-0·340 to 0·376) | 0·919 |
| Descending thoracic aorta | | | | | |
|  | SUV_max_ | 0·281±0·316 | 0·268±0·473 | 0·013 (-0·250 to 0·275) | 0·920 |
|  | TBR_max_ | 0·851±0·415 | 0·856±0·418 | -0·005 (-0·275 to 0·265) | 0·970 |
|  | cSUV_max_ | -1·089±0·684 | -1·115±1·025 | 0·027 (-0·542 to 0·595) | 0·925 |
|  | MDS SUV_max_ | 0·618±0·334 | 0·717±0·438 | -0·099 (-0·348 to 0·150) | 0·426 |
|  | MDS TBR_max_ | 1·156±0·450 | 1·306±0·443 | -0·148 (-0·450 to 0·154) | 0·328 |
| Abdominal aorta (non-aneurysmal) | | | | | |
|  | SUV_max_ | 0·762±0·316 | 0·726±0·366 | 0·037 (-0·186 to 0·259) | 0·741 |
|  | TBR_max_ | 1·333±0·532 | 1·314±0·489 | 0·018 (-0·313 to 0·350) | 0·911 |
|  | cSUV_max_ | -0·104±0·652 | -0·1058±0·556 | 0·002 (-0·392 to 0·397) | 0·991 |
|  | MDS SUV_max_ | 1·158±0·363 | 0·995±0·369 | 0·163 (-0·724, to 0·399) | 0·170 |
|  | MDS TBR_max_ | 1·697±0·537 | 1·583±0·510 | 0·114 (-0·221 to 0·449) | 0·494 |
| Abdominal aorta (AAA) | | | | | |
|  | SUV_max_ | 1·142±0·283 | 0·726±0·366 | 0·416 (0·203 to 0·629) | <0·0001 |
|  | TBR_max_ | 1·712±0·560 | 1·314±0·489 | 0·398 (0·057 to 0·739) | 0·023 |
|  | cSUV_max_ | 0·543±0·432 | -0·1058±0·556 | 0·649 (0·324 to 0·973) | <0·0001 |
|  | MDS SUV_max_ | 1·427±0·356 | 0·995±0·369 | 0·432 (0·197 to 0·668) | 0·001 |
|  | MDS TBR_max_ | 1·997±0·568 | 1·583±0·510 | 0·414 (0·064 to 0·764) | 0·022 |
|  | Agatston score | 11·444±1·760 | 7·338±3·811 | 4·105 (2·013 to 6·198) | 0·001 |

Values are logarithm base 2 transformed and presented as mean±standard deviation

AAA, abdominal aortic aneurysm; ^18^F-NaF, ^18^F-sodium fluoride; MDS, most-diseased segment; CI, confidence interval

**Supplemental Table 2**

Multivariable Analysis of ^18^F-Sodium Fluoride Uptake and Clinical Outcomes

|  | **Increase in Expansion**  **(mm/year; 95% CI)** | **P-value** | **Hazard Ratio (95% CI) for Composite AAA Events** | **P-value** |
| --- | --- | --- | --- | --- |
| Model 1 | 0·365 (0·34 to 1·90) | 0·006 | 2·16 (1·03 to 4·51) | 0·041 |
| Model 2 | 0·375 (0·39 to 1·91) | 0·004 | 2·26 (1·97 to 4·76) | 0·033 |
| Model 3 | 0·259 (0·029 to 1·56) | 0·042 | 2·49 (1·07 to 5·78) | 0·034 |
| Model 4 | 0.357 (0.315-1.880) | 0.007 | 2.096 (1.004-4.375) | 0.049 |
| Model 5 | 0.346 (0.295-1.834) | 0.008 | 2.103 (1.002-4.412) | 0.049 |
| Model 6 | 0.267 (0.052-1.589) | 0.037 | 2.162 (0.970-4.819) | 0.059 |
| Model 7 | 0.266 (0.026-1.597) | 0.041 | 2.194 (0.955-5.028) | 0.064 |

Expansion rate was logarithm base 2 transformed

Model 1: unadjusted

Model 2: adjusted for age, sex

Model 3: adjusted for age, sex, baseline diameter, body-mass index, systolic blood pressure, smoking

Model 4: adjusted for diastolic blood pressure

Model 5: adjusted for diastolic blood pressure, eGFR

Model 6: adjusted for age, sex, body-mass index, diastolic blood pressure, smoking, diameter, eGFR

Model 7: adjusted for age, sex, body-mass index, diastolic blood pressure, smoking, diameter, eGFR, peripheral arterial disease

AAA, abdominal aortic aneurysm; CI, confidence intervals; eGFR, estimated glomerular filtration rate
